# Increased Leptin Response and Inhibition of Apoptosis in Thymocytes of Young Rats Offspring from Protein Deprived Dams during Lactation

**DOI:** 10.1371/journal.pone.0064220

**Published:** 2013-05-10

**Authors:** Simone Vargas da Silva, Carolina Salama, Mariana Renovato-Martins, Edward Helal-Neto, Marta Citelli, Wilson Savino, Christina Barja-Fidalgo

**Affiliations:** 1 Departamento de Biologia Celular, Instituto de Biologia, Universidade do Estado do Rio de Janeiro, Rio de Janeiro, RJ, Brazil; 2 Departamento de Nutrição Básica e Experimental, Instituto de Nutrição, Universidade do Estado do Rio de Janeiro, Rio de Janeiro, RJ, Brazil; 3 Laboratório de Pesquisas sobre o Timo, Instituto Oswaldo Cruz, Fundação Oswaldo Cruz, Rio de Janeiro, RJ, Brazil; Federal University of São Paulo (UNIFESP), Escola Paulista de Medicina, Brazil

## Abstract

We investigated the consequences of mild maternal malnutrition in rat dams, in terms of thymocyte responses and the putative role of leptin. The young progeny of dams submitted to protein deprivation (PD) during lactation showed at 30 days of age lower body and thymus weights, significant alterations in CD4/CD8-defined T cell subsets without modifications in total thymocyte number as well as in proliferative response. Despite, the rats from PD group did not present alterations in leptin circulating levels, the expression of leptin receptor ObRb was enhanced in their thymocytes. This change was accompanied by an increase in leptin signaling response of thymocytes from PD rats, with an increase in JAK2 and STAT3 phosphorylation after leptin stimulation. Thymocytes from PD rats also presented a decreased rate of spontaneous apoptosis when compared to controls. Accordingly, higher expression of anti-apoptotic protein Bcl-2, and lower of pro-apoptotic protein Bax, with no change of pro-apoptotic Bad, and higher pro-caspase 3 content were detected in PD thymocytes. Moreover, thymocytes from PD group exhibited a constitutive higher nuclear content of p65 NF-kB associated to a lower IkB content in the cytoplasm. Finally, although there was no change in *ob* gene expression in PD thymocytes, a higher mRNA expression for the *Ob* gene was observed in the thymic microenvironment from PD animals. Taken together, the results show that mild maternal protein deprivation during lactation affects thymic homeostasis, enhancing leptin activity, which in turn protects thymocytes from apoptosis in the young progeny, with possible consequences upon the immune response of these animals in adult life.

## Introduction

Epidemiological and experimental studies have demonstrated that maternal nutritional imbalance and metabolic disturbances during critical time windows of development may have a persistent effect on the offspring’s health [1]. In particular, it has been demonstrated that nutritional deficiency during early life alters crucial physiological circuits, with changes in hormone receptors [2], [3], signaling molecules [4]–[8] and regulatory enzymes [4], [8], [9]. In rats, we have reported that adult offsprings from dams submitted to protein deprivation during early lactation exhibit an imbalance in insulin and glucocorticoid secretion, which affects the proper development of acute inflammatory responses. This was ascertained by lower leukocyte migration into inflammatory sites, decreased edema formation, and lower expression of the adhesion molecule ICAM1 [10], [11].

Maternal malnutrition during early lactation also affects the expression and activation of key proteins of the insulin signaling cascade, leading to a permanent up-regulation of PI3K, AKT and increase in GLUT4 translocation in skeletal muscle and adipocytes from adult offspring [6], [8]. Besides interfering with insulin-mediated responses, maternal protein restriction during lactation interferes with leptin secretion in offsprings, which present higher serum leptin contents and increased leptin receptor expression in the pituitary gland [12], [13].

Leptin is a hormone/cytokine produced mainly by adipocytes in direct proportion to whole-body fat mass [14]. It regulates the energy availability in peripheral adipose tissue through specific hypothalamic signals and affects body weight, food intake, body temperature and metabolic rate [14]. Interestingly, it has been shown that leptin is also produced by the pituitary gland, skeletal muscle, placenta, stomach, thymus and mammary gland [15]–[22].

Accumulating evidence supports a role for leptin in the regulation of adaptive immunity, at least in part, by the direct action of the hormone on T cells [23], [24]. Accordingly, db/db mice exhibit a precocious thymic atrophy with significant decrease in the secretion of the thymic hormone thymulin, by the thymic epithelium [25], [26]. Moreover, ob/ob and db/db mice exhibit increased susceptibility to infection and decreased Th1-type responses.

Likewise, the reduction in plasma leptin levels observed for humans and rodents subjected to caloric restriction correlates with T cell deficiency, impaired adaptive immune function, and markedly increased susceptibility to infection [27]–[29].

Leptin is also able to inhibit thymic apoptosis in young rats, and this effect seems to be dependent on the expression of the long form of the ObR, but not on the activation of the receptor associated tyrosine-kinase JAK2 [30].

Herein we studied the effect of mild maternal protein malnutrition during lactation on thymocytes of young adult offspring, as well as the role of the leptin on the expression of cell death associated molecules.

## Materials and Methods

### Ethics Statement

This study was carried out in strict accordance with the recommendations in the Guide for the Care and Use of Laboratory Animals of the National Institutes of Health. The protocol was approved by the Committee on the Ethics of Animal Experimental of the Instituto de Biologia Roberto Alcantara Gomes from Universidade do Estado do Rio de Janeiro (Permit number: CEA/047/2009).

### Animals and Diet Protocol

Wistar rats obtained from animal facilities of the Oswaldo Cruz Foundation (Rio de Janeiro, Brazil) were housed in a controlled room (25±1°C) at 60% humidity and were maintained in an artificial dark-light cycle (light from 7∶00 a.m. to 7∶00 p.m.).

Three-month-old virgin female rats were mated, and pregnant dams, housed in individual cages, were fed *ad libitum* during gestation with a normal isocaloric diet containing 22% protein. Following delivery, each lactating dam was paired with 6 male pups and separated into two groups. The first group of dams received a protein-restricted diet (PRD), containing 8% protein during all lactation, whereas the second group was pair-fed with a normal diet containing 22% protein (NPD). Both diets were isocaloric and contained the same amount of vitamins and mineral salts, as seen in Table 1 [31].

**Table 1:** Composition of control and protein-restricted (8%) diet.

	Control diet^a^	Protein-restricted diet^b^
**Ingredients (g/Kg)**		
Soybean+wheat	230.0	80.0
Corn starch	676.0	826.0
Soybean oil	50.0	50.0
Vitamin mix^c^	4.0	4.0
Mineral mix^c^	40.0	40.0
**Macronutrient composition (%)**		
Protein	23.0	8.0
Carbohydrate	66.0	81.0
Fat	11.0	11.0
Total energy (Kj/Kg)	17038.7	17038.7

a Standard diet for rats (Nuvilab – NUVITAL Nutrientes LTDA, Paraná, Brazil).

b The protein restricted diet was prepared in our laboratory using the control diet and replacing part of its protein with cornstarch. The amount of the latter was calculated so as to make up for the decrease in energy content due to protein restriction.

c Vitamin and mineral mix were formulated according to the American Institute of Nutrition 93-G recommendation for rodents diets [31].

At the end of lactation (21 days post natally), offsprings from each group were housed in separate cages and subdivided in two groups, namely ***Controls*** (**C**: offsprings from mothers that had received the NPD) and ***Protein-Deprived*** (**PD**: offsprings from mothers that received the PRD during the lactation). Both groups were then fed with a normal diet *ad libitum* until they were 30 days old. For all the experiments, young rats (between 28 and 32 days old) formed the C and PD matched-groups. In order to avoid litter effects, the adult animals used in all experiments were taken randomly from at least three different litters. The body weight of all animals was evaluated on the day of the experiments.

### Reagents

Leptin was acquired from Calbiochem (La Jolla, CA). RPMI, Trizol reagent and reagents for cell culture were purchased from Invitrogen Corp (Carsbad, CA). Ficcol Hypaque, PVDF membranes (Hybond – P) and rainbow markers were purchased from Amersham Biosciences (Buckinghamshire, UK) and streptavidin from Caltag Laboratories.

Antibodies with specificity to the ObR (sc 8391), JAK2 (sc 294), pJAK2 (sc 21870), STAT3 (sc 8019), pSTAT3 (sc 8059), IkB (sc 371), NF-kB (sc 372), BAD (sc 7869), BCL2 (sc 783), BAX (sc 6236), CASPASE3 (sc 7148), GR (sc 8992), HISTONE (sc 8654) and ACTIN (sc 8432) were from Santa Cruz Biotechnology (Santa Cruz, USA). Phycoeritrin (PE)-conjugated mouse anti-CD4, and fluorescein isothiocyanate (FITC)-coupled anti-CD8 monoclonal antibodies were from Biolegend (San Diego, USA).

Sense and antisense oligonucleotides specific for the Ob and ObRb were produced by Prodimol Biotechnology (Belo Horizonte, MG). Unless otherwise stated, all other reagents were obtained from Sigma-Aldrich (Saint Louis, USA).

### Isolation of Thymocytes and the Thymic Microenvironment

Isolated thymocytes were obtained from **control** and **PD** rats. Briefly, after 12 h fast, rats were sacrificed in the CO_2_ chamber; thymuses were obtained, weighted and gently dissociated in incomplete RPMI through steel net. After dissociation, the thymic microenvironment retained on the steel net was used for PCR analysis and the cell suspension was layered over Ficcol Hypaque and centrifuged. Thymocytes thus isolated were resuspended in RPMI containing penicillin, streptomycin and 10% (v/v) fetal calf serum and incubated to 37°C for 1–2 hours to adherence of macrophages. Under all experimental conditions, >97% of cells were viable as assessed by trypan blue dye exclusion. Total counting of thymocytes was made in Neubauer chambers.

### Immunoblotting

To obtain the total cell extracts, isolated thymocytes (5×10^6^ cells/mL) were lysed and proteins were extracted in 50 nM Hepes, 1 mM MgCl_2_, 10 mM EDTA (pH = 6,4), 1% (v/v) Triton X-100, 2 mM Sodium Orthovanadate, 1 µg/mL DNase, 0.5 µg/mL RNase containing the following protease inhibitors: 1 mM PMSF, 1 µg/mL leupeptin, 1 µg/mL aprotinin and 1 µg/mL soybean trypsin inhibitor.

Nuclear extracts were obtained as described earlier [32]. Briefly, isolated thymocytes (5×10^6^ cells/mL) were lysed in ice-cold buffer A (10 mM HEPES, pH 7.9, 10 mM KCl, 0.1 mM EDTA, 0.1 mM EGTA, 1 mM DTT, and 1 mM PMSF, 1 µg/mL pepstatin, 1 µg/mL aprotinin and 1 µg/mL leupeptin), and after a 15-min incubation on ice, Nonidet P-40 was added to a final concentration of 0.5% (v/v). Nuclei were collected by centrifugation (1,800 g, 5 min at 4°C). The nuclear pellet was suspended in ice-cold buffer C (20 mM HEPES, pH 7.9, 400 mM NaCl, 1 mM EDTA, 1 mM EGTA, 1 mM DTT, 1 mM PMSF, 1 µg/mL pepstatin, 1 µg/mL aprotinin, 1 µg/mL leupeptin, and 20% (v/v) glycerol) and incubated for 30 min. Nuclear proteins were harvested in the supernatant after centrifugation (13,000 g; 10 min at 4°C).

Total protein content of cell extracts and nuclear extracts were determined by Bradford’s method [33]. Samples were resolved to SDS-PAGE and proteins were transferred to PVDF membranes. Rainbow markers were run in parallel to estimate molecular weights. Membranes were blocked with Tween-PBS (0.1% Tween-20) containing 5% bovine serum albumin and incubated with a specific primary antibodies: anti-ObR (1∶1000); anti-IkB (1∶1000); anti-NF-kB (1∶1000); anti-BAD (1∶500); anti-BCL2 (1∶500); anti-BAX (1∶500); anti-CASPASE3 (1∶1000); anti-JAK2 (1∶1000); anti-pJAK2 (1∶1000); anti-STAT3 (1∶1000); anti-pSTAT3 (1∶1000); anti-GR (1∶1000). After extensive washing in Tween-PBS, PVDF sheets were incubated with the appropriate secondary biotin-conjugated antibody (1∶10000) (Santa Cruz Biotechnology) for 1 h and then incubated with horseradish peroxidase-conjugated streptavidin (1∶10000). Immunoreactive proteins were visualized using the ECL system. Membranes were stripped with stripping buffer (62.5 mM Tris-HCl, 2% SDS and 100 mM β-mercaptoethanol) and re-probed similarly with anti-actin or anti-histone antibody (1∶1000). Films were scanned and semi-quantitatively analyzed. The cellular extracts and nuclear extracts were normalized to actin and histone levels respectively; the bands being quantified by densitometry, using ImageJ 1.34 s Software (NIH, USA).

### Leptin and Corticosterone Measurements

Animals were anesthetized with Ketamine (50 mg/Kg) and xylazine (20 mg/Kg). Blood was collected by cardiac puncture and serum levels of leptin and corticosterone were measured using appropriate ELISA kits (Peprotech, Rocky Hill, NJ and Cayman–Ann Arbor, MI; respectively), as precluded by the manufacturers.

### Thymocyte Phenotyping, Subpopulation Sorting and Evaluation of Apoptosis

Thymocyte suspensions (1.0×10^7^/mL) in binding buffer were incubated for 20 min in the dark, at room temperature, with 100 µL of the following panel of antibodies PE-CD4/FITC-CD8. Thereafter, cells were washed with binding buffer. A two-color analysis was made using a BD FACSAria Cell Sorting System (Becton Dickinson Biosciences, San Jose, CA) and analyzed using CellQuest software (Becton Dickinson Biosciences).

CD4^−^CD8^−^ (double negative - DN), CD4^+^CD8^+^ (double positive - DP), as well as CD4^+^ and CD8^+^ single-positive T cells were analyzed and sorted from a suspension of whole thymocytes by multiparameter, cell sorting BD FACSAria Cell-Sorting device (Becton Dickinson Biosciences, San Jose, USA).

Thymocytes (1.0×10^6^/mL) isolated as described above, were also tested for apoptosis using the annexin V technique [34]. Cell suspensions were washed with binding buffer (10 mM HEPES, pH 7.4; 150 mM NaCl; 5 mM KCl; 1 mM MgCl2; 1.8 mM CaCl) and incubated for 20 min with 100 µL of Annexin V-FITC. After incubation, cells were stained with propidium iodide (PI - 100 µg/mL) for necrosis exclusion. PI and annexin V binding were assessed by flow cytometry. A total of 10,000 cells were acquired. Unlabeled cells suspended in binding buffer were used as negative controls and for the determination of gates to be used in the apoptosis assay. Apoptotic cells were measured in the annexin V-positive propidium iodide-negative quadrant and divided by the total number of cells in the gated region.

### Isolation of Spleen Cells

Isolated spleen cells were obtained from **control** and **PD** rats. Briefly, after 12 h fast, rats were sacrificed in the CO_2_ chamber; spleens were obtained and gently dissociated in incomplete RPMI. After dissociation, the cell suspension was layered over Ficcol Hypaque and centrifuged. Spleen cells thus isolated were resuspended in RPMI containing penicillin, streptomycin and 10% (v/v) fetal calf serum and incubated to 37°C for 1–2 hours to adherence of macrophages. Under all experimental conditions, >97% of cells were viable as assessed by trypan blue dye exclusion. Total counting of thymocytes was made in Neubauer chambers.

### Spleen Cells Phenotyping

Spleen cells suspensions (1.0×10^6^/mL) in binding buffer were incubated for 20 min in the dark, at room temperature, with 100 µL of the following panel of antibodies PE-CD4/FITC-CD8. Thereafter, cells were washed with binding buffer. A two-color analysis was made using a BD FACScalibur System (Becton Dickinson Biosciences, San Jose, CA) and analyzed using CellQuest software (Becton Dickinson Biosciences). CD4−CD8− (double negative - DN), CD4+CD8+ (double positive - DP), as well as CD4+ and CD8+ single-positive T cells were analyzed from a suspension of whole cells. A total of 10,000 cells were acquired. Unlabeled cells suspended in binding buffer were for the determination of gates and the isotypes controls PE-IgG and FITC-IgG were used as negative controls.

### Thymocyte Proliferation

Thymocyte proliferation was evaluated by [H^3^]-thymidine incorporation [35]. Briefly, isolated thymocytes from **control** and **PD** rats (1.0×10^6^ cells/mL) were incubated onto 96-well plates in RPMI supplemented with 10% (v/v) fetal calf serum, 100 µg/mL penicillin and 100 U/mL streptomycin, and stimulated for 24 hours with Concanavalin A (2 µg/mL) or Leptin (10 ηg/mL) or Leptin+ConA, at 37°C in an atmosphere of 5% CO_2_. Proliferation was determined by liquid scintillation counting after the addition of 1 µCi/well [H^3^]-thymidine in the last 6 hours of incubation. The dose of leptin used in our experiments was based in data of literature [30].

### Quantitative Gene Expression in Thymocyte Subsets

Total RNA was extracted from DN, DP, CD4 and CD8 cells, or from the thymic microenvironment, using TRIzol reagent according to the instructions of the manufacturer, and quantified by spectrophotometry. After DNase treatment (RQ1 RNase-Free DNase; Promega, São Paulo, Brazil), total RNA (2.0 µg) was reverse transcribed using moloney murine leukemia virus reverse transcriptase and oligo (dT) 15 primer. Quantitative real time PCR was performed in a Rotor gene Q using a SYBR-green fluorescence quantification system (Qiagen) to quantify amplicons. The standard PCR conditions were 95° for 5 minutes, then 35 cycles at 95°C (5 s) and 60°C (10 s) followed by the standard denaturation curve. Primers based on the sequence of Rattus norvegicus *Ob* (Gene bank accession n°: NM_013076.3) were used to amplify a 81 bp *Ob* cDNA: sense: 5′-TCCTGTGGCTTTGGTCCTAT-3′ and antisense: 5′-TGATGAGGGTTTTGGTGTCA-3′; Rattus norvegicus *Obr* (Gene bank accession n°: AF_287268) were used to amplify a 132 bp *Obr* cDNA: sense: 5′-CTGCTGGAGTCCCAAACAAT-3′ and antisense: 5′-CATTCCCAAAGCAACAGTGG-3′; *Gapdh* primers were used to validate the cDNA (Gene bank accession n°: DQ_403053) in each reaction: sense: 5′-TCAACGGGAAACCCATCACCATCT-3′ and antisense: 3′-ACGACATACTCAGCACCAGCATCA-3′.

### Statistical Analysis

The data were expressed as means±standard error and analyzed by the two-tailed unpaired Student’s *t* test or analysis of variance (ANOVA). When appropriate, individual comparisons were subsequently tested with Bonferoni’s t test for unpaired values. Differences were considered statistically significant when *p≤*0.05. The data were analyzed using GraphPad Prism version 5.00 for Windows (GraphPad Software, USA).

## Results

### Maternal Protein Deprivation Affects Body Weight but not Thymus Cellularity in the Offsprings

We have first investigated whether mild maternal protein deprivation (8% protein diet) during lactation affects the thymus and body weights of the young offspring at 30^th^ day after birth. Table 2 shows that the body weight of young rats from the PD group is lower (∼37%) compared to controls. Maternal protein deprivation during lactation also affected the thymus weight of offsprings, with a significant reduction (∼27%) compared to controls. In contrast, no differences were detected in the thymus/body weights ratio, in the total number of thymocytes, or in the number of thymocytes per mg tissue, suggesting that the maternal protein restriction model applied did not cause severe damage in the offspring thymus cellularity.

**Table 2:** Maternal protein deprivation does not alters the relative numbers of thymocytes in young rats.

	Control	Protein Deprivation
Body weight (g)	95.83±2.81	60.36±2.58*
Thymus weight (g)	0.41±0.01	0.30±0.02*
Relative thymus weight (g/g)	0.0046±0.0004	0.0044±0.0003
Total number of thymocytes (×10^8^)	4.03±0.33	3.42±0.29
Total number of thymocytes/thymus weight (×10^5^ cells/mg of thymus)	0.98±0.08	1.08±0.09

Results are expressed as means ± SEM; n = 9–16.

* P<0.05.

### Maternal Protein Deprivation Results in Changes in the Percentages of CD4/CD8-defined Thymocyte Subsets

**Figure 1:**
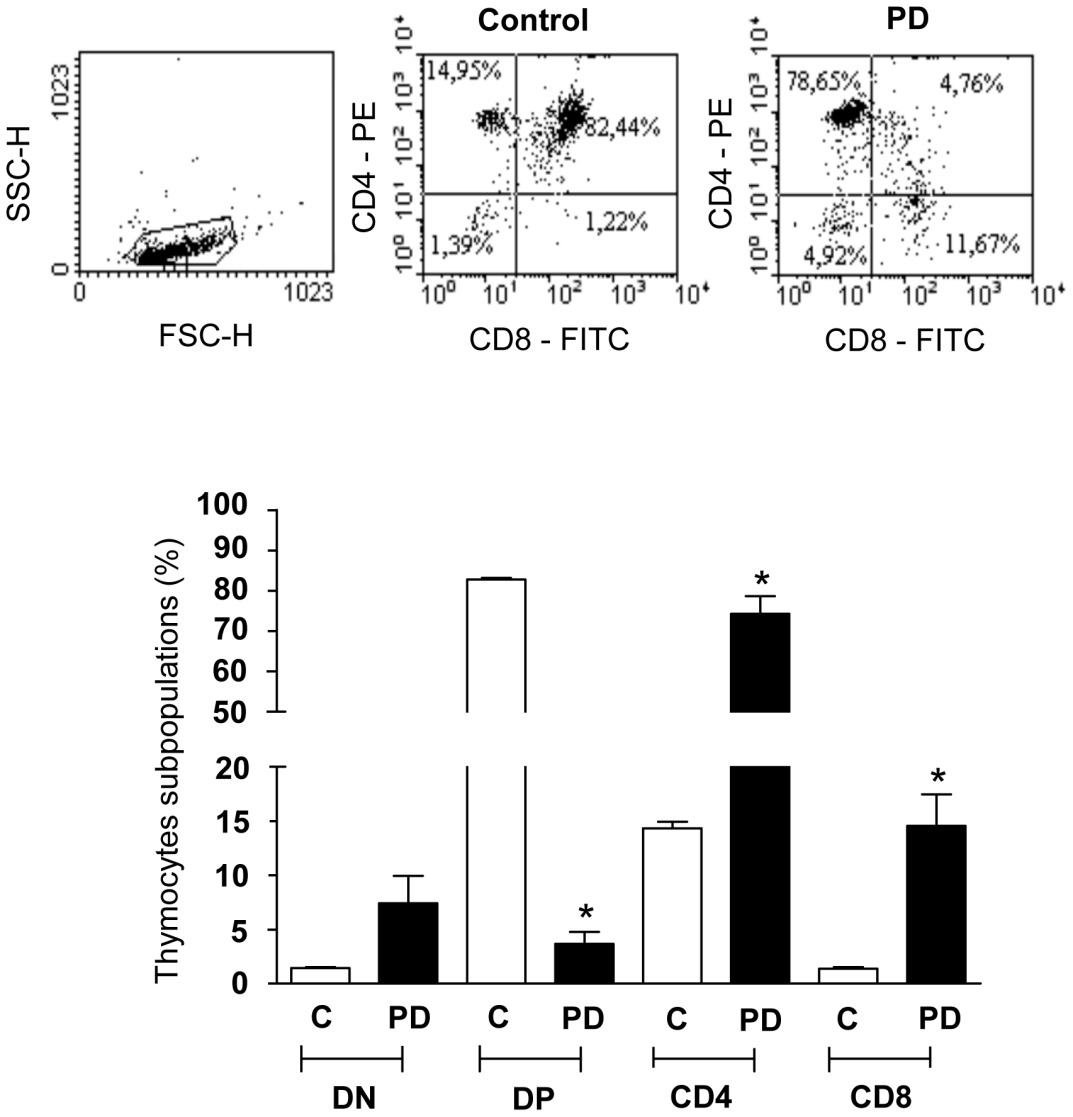
Maternal protein deprivation during lactation modulates the relative numbers of CD4/CD8-defined thymocyte subsets in young offspring. Panel A depicts representative flow cytometry analysis of proportions of CD4^+^, CD8^+^, CD4^+^CD8^+^ and CD4^−^CD8^−^ T cells from control and PD animals. Values within each quadrant correspond to the means±standard error of 6 rats per group. *p<0.05, compared PD to respective control.

To investigate the hypothesis that mild maternal protein deprivation affects the maturation of thymocytes from the young adult offsprings, we evaluated the expression of membrane surface markers in these cells, isolated from control and PD groups. We showed that thymus isolated from PD rats presented higher percentages of mature CD4^+^ and CD8^+^ single-positive thymocyte subsets, when compared to the control group. In addition, there was a reduction of CD4^+^CD8^+^ T cell population in the PD animals. However, there was no difference between control and PD groups, in the percentages of the most immature CD4^−^CD8^−^ T cell subpopulation (Fig. 1). We also evaluated in spleen cells the expression of these membrane surface markers. As showed in the table 3, we did not observed alteration in the percentages of CD4^−^CD8^−^, CD4^+^CD8^+^, CD4^+^ and CD8^+^ single-positive T cells in the PD animals.

**Table 3:** Maternal protein deprivation does not alter the expression of membrane surface markers of spleen cells in young rats.

	*Control*	*Protein Deprivation*
**CD4** ^−^ **CD8** ^−^	59.7±5.54%	61.4±0.95%
**CD4^+^CD8^+^**	2.37±0.42%	2.8±1.06%
**CD4^+^**	30.07±9.17%	23.8±4.04%
**CD8^+^**	10.8±1.04%	11.97±3.61%

Results are expressed as means ± SEM; n = 3–5.

### Normal Serum Leptin Levels in 30-days Old Rats Exposed to Protein Deprivation during Lactation

**Figure 2:**
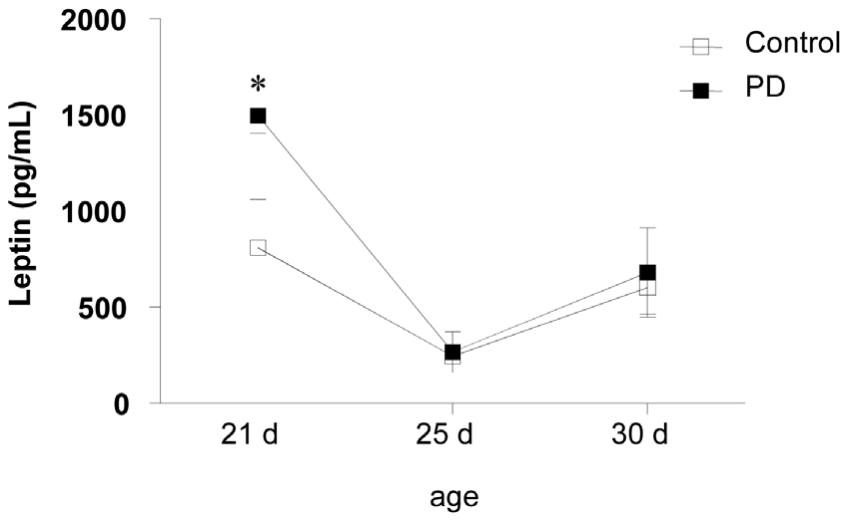
Maternal protein deprivation during lactation does not affect serum leptin concentration from 25 and 30-day old offspring. Blood samples were collected from control and PD animals at 21, 25 and 30 days of age, and leptin serum levels were determined by ELISA. Results are expressed as means ± S.E of 8–12 animals/group. *P<0.05, compared to the control group.

Serum leptin levels were evaluated in rats from PD and control groups. By three weeks after birth, leptin’s levels were raised in PD rats when compared to controls groups confirming previous data [12]. In 25 and 30-day-old animals, the differences in the serum leptin levels between the groups tested were no longer detected (Fig. 2). These findings indicated that, by day 25 and 30 of age, leptin-controlling homeostatic circuit operates normally.

### Leptin Signaling Pathways in Thymocytes of PD Rats

**Figure 3:**
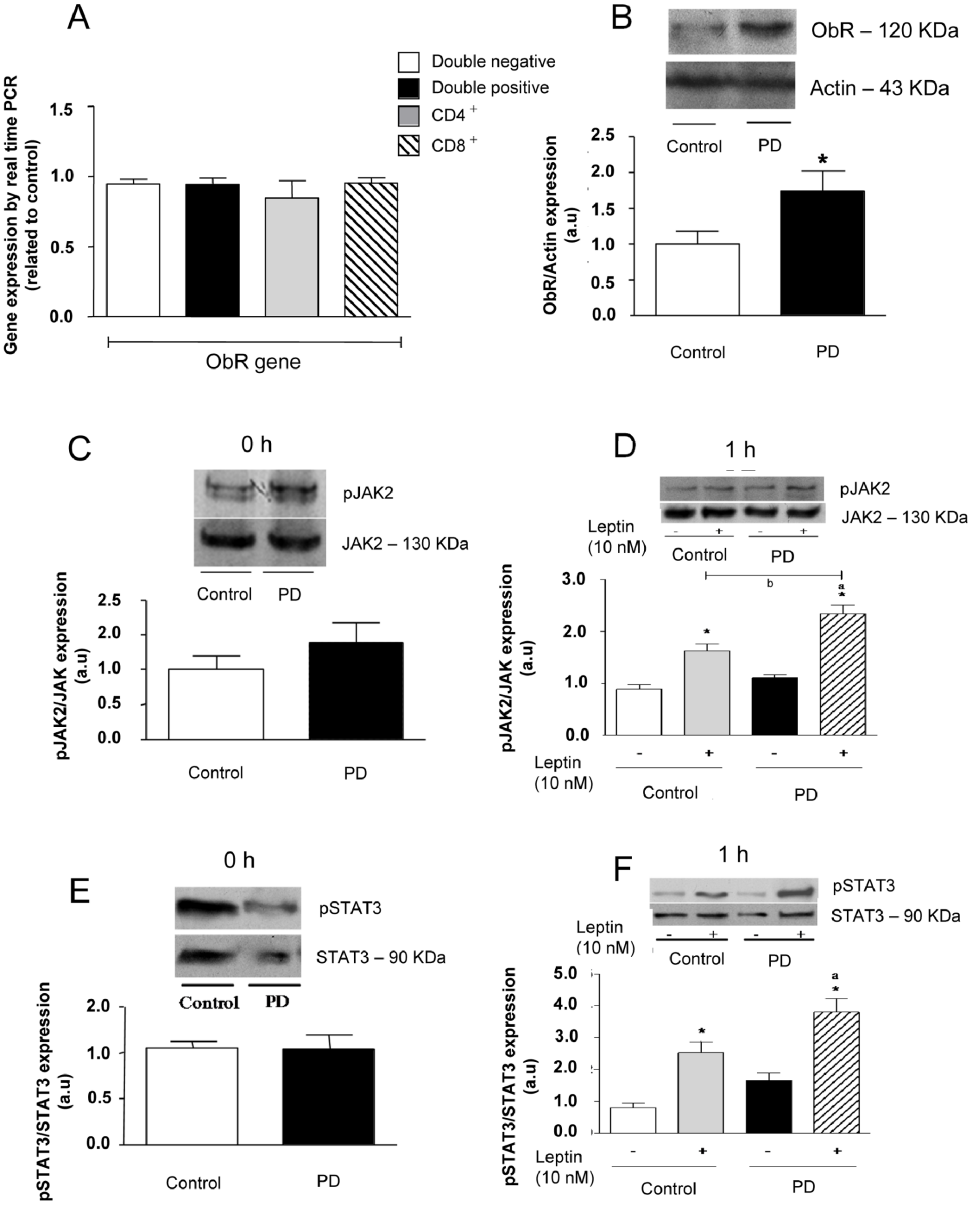
Thymocytes from PD rats present a higher ObR protein expression in basal conditions, and increase JAK-2 and STAT-3 activation after leptin stimulation. Panel A shows that ObRb gene expression levels are similar in all CD4/CD8-defined thymocyte subsets from both control and PD rats. Total RNA was harvested and analyzed by qRT-PCR for ObRb mRNA. The result was normalized by GAPDH, and data were expressed as gene expression levels related to control. Panel B depicts immunoblottings showing that the ObR protein contents in thymocytes from PD animals were significantly higher than the control counterparts (*p<0.05). Quantitation of bands was expressed in arbitrary units related to actin expression, applied as internal control of the experiment. Values are means ± S.E of 6–10 animals/group. No differences in the pJAK-2/total JAK-2 ratios were seen in control or PD thymocytes, in basal conditions (panel C). Incubation with leptin significantly enhanced pJAK-2/total JAK-2 ratios in both groups, with the increase being significantly more prominent in the PD group (Panel D). Similar data were recorded for the expression of pSTAT-3 versus total STAT-3 in basal and leptin conditions, as seen in panels **e** and **f**. Thymocyteswere incubated in the presence or absence of leptin (10 nM) for 1 hour and pJAK-2 and pSTAT3 protein expression were assessed in total extracts by immunoblotting. Quantification of bands is expressed in arbitrary units. Values are means ± S.E of 6–10 animals/group. *P<0.05 compared to control group; ^a^p<0.05 compared to PD group and; ^b^P<0.05 compared to control+Leptin group.

Since the most leptin actions are delivered through the activation of Ob receptor (ObR), we evaluated the expression of this receptor as well as the corresponding intracellular signaling pathways on thymocytes isolated from PD and control rats. No differences were detected in the mRNA expression of the *Obrb* gene in purified CD4/CD8-defined T cell subpopulations (Fig. 3A).

While no changes were observed in the mRNA for *Obrb* gene in the T cells subpopulations, we evaluated the expression of ObR in whole thymocytes by western-blotting. As shown in Fig. 3B thymic cells isolated from PD rats showed an enhanced expression level of this receptor when compared to controls. These results suggest a potential response to leptin signaling in thymocytes from PD group.

We also evaluated the activation of the JAK2/STAT3 signaling pathway in these cells. In the absence of leptin, no significant difference was observed in the contents of pJAK2 and pSTAT3 (Fig. 3C–E) in thymocytes isolated from either PD or control groups. However, the treatment of thymic cells with leptin for 1 hour enhanced the phosphorylation of JAK2 (control+Lep: ∼1.5 *versus* PD+Lep: ∼2.0-fold) and STAT3 (control+Lep: ∼2.0 *versus* PD+Lep: ∼3.2-fold) in both groups, although these increases were higher in PD group (Fig. 3D and 3F, respectively).

### Decrease in Spontaneous Apoptosis of Thymocytes from PD Rats

**Figure 4:**
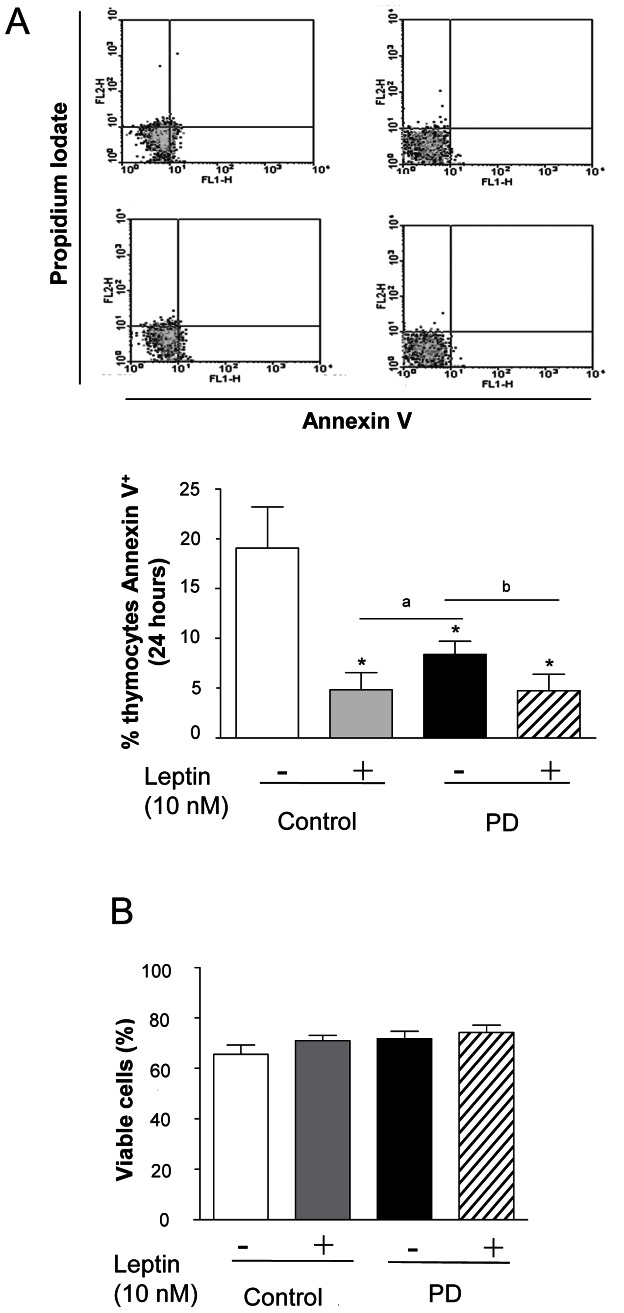
Thymocytes from PD rats present inhibition of baseline apoptosis after 24 hour of incubation. (A) Apoptosis of thymocytes was evaluated by determination of annexin V expression by flow cytometry. (B) Thymocytes from C and PD group were incubated in the presence or absence of leptin (10 nM) for 24 h. After incubation, viable cells were counted in Neubauer chambers. Results are expressed as means ± S.E of 6–10 animals/group. *P<0.05 compared to control group; ^a^p<0.05 compared to PD group and; ^b^P<0.05 compared to control+Leptin group.

We also evaluated the early apoptotic events by measuring the exposure of phosphatidylserine on plasma membranes by flow cytometry, using annexin V-FITC plus PI. Thymocytes isolated from PD and control groups were incubated for 24 hours in the presence or absence of 10 nM leptin. As shown in fig. 4A, in basal conditions, there was a lower Anexin V-FITC staining in the PD cells when compared to controls (∼58%). In addition, the incubation with leptin (24 h) clearly protected the thymocytes from apoptosis in both groups.

To confirm the above data, we ascertained the viability of the cells by trypan blue dye exclusion. After 24-h incubation, no difference in cell viability was observed between thymocytes isolated from control and PD rats, in the presence or absence of leptin 10 nM (Fig. 4B).

**Figure 5:**
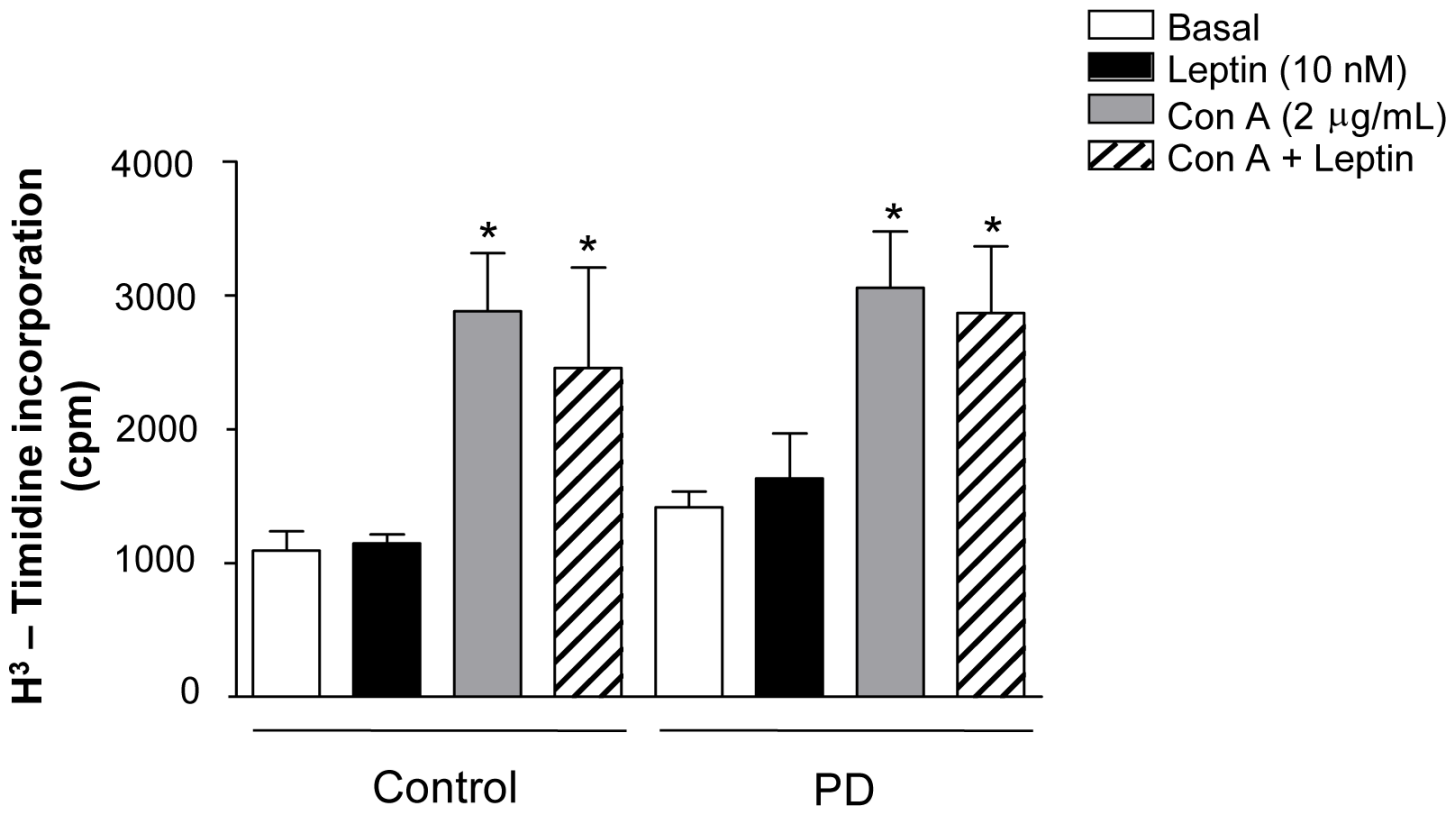
Maternal protein deprivation during lactation does not affect the thymocyte proliferation from young offspring. Thymocytes were cultured with the indicated stimulus for 24 hours. [H^3^] thymidine was pulsed for the last 6 hours. Results are expressed as means±SE of 6–12 animals/group. *P<0.05 compared to respective basal condition.

Because the higher rate of thymic cell survival could be related to an increased rate of proliferation, we assessed the proliferative response in PD and control thymocytes, incubated for 24 h with or without 10 nM leptin. In baseline conditions, we observed no difference between the groups. Furthermore, leptin did not alter the proliferative response in thymocytes from any groups. As expected, stimulation with the T cell mitogen concanavalin A for 24 hours markedly increased the proliferation in both groups (about 3-fold). The simultaneous incubation with leptin did not interfere in the proliferative capacity induced by concanavalin A in any groups (Fig. 5).

### Differential Expression Levels of Pro- and Anti-apoptotic Protein Expression in PD Rat Thymocytes

**Figure 6:**
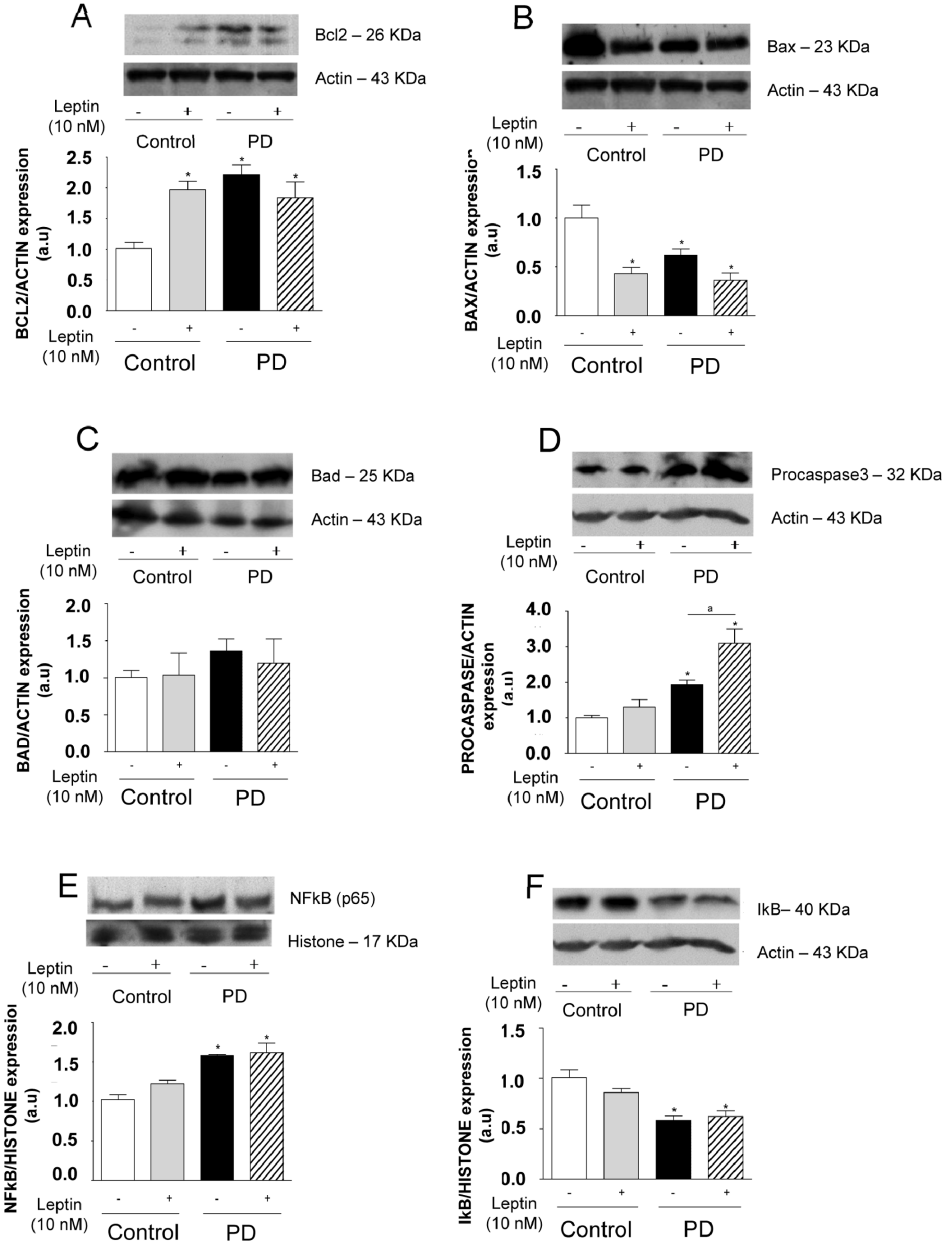
Thymocytes from PD rats present alterations in the pro- and anti-apoptotic protein levels and NF-kB nuclear translocation in basal condition. Thymocytes isolated from control and PD rats were incubated in the presence or absence of leptin (10 nM) for 24 hours, and BcL-2, Bad, Bax, procaspase 3, NF-kB, IkB, actin and histone protein expression were assessed in total (BcL-2, Bad, Bax, procaspase3, IkB and actin) or nuclear (NF-kB and histone) extracts by western blotting analysis. Quantification of bands is expressed in arbitrary units. Values are means ± S.E of 6 animals/group. *P<0.05 compared to control group.

We also evaluated the expression of anti-apoptotic (BCL2) and pro-apoptotic (BAD and BAX) and PROCASPASE3 proteins in thymocytes isolated from control and PD groups, 24 h after leptin incubation. As seen in fig. 6A, in basal conditions, thymocytes isolated from PD rats presented higher levels of BCL2 as compared to controls (∼100%). In these conditions, the expression of BAX was diminished (∼57%) and we observed no significant difference in the BAD expression between the groups. Furthermore, we detected an up-regulation of PROCASPASE3 (∼93%), suggesting that CASPASE3 is predominantly in its inactive form.

### NF-kB Pathway Activation in Thymocytes from PD Rats

NF-kB is a transcription factor present in the majority of cells. It had been described a direct involvement of the NF-kB pathway in cell survival signals expression, including its ability to induce BCL2 [36]. We next investigated the effects of maternal protein restriction during lactation on the activation of NF-kB in thymocytes from young offspring. To address this, the nuclear content of the p65 NF-kB subunit in PD thymocytes was determined by Western blotting. As shown in figure 6D, thymocytes from the PD group (after 24 h of incubation), exhibited a constitutive high nuclear content of NF-kB p65 subunit (about 50% of increase), when compared with thymocytes from control counterparts. Accordingly, at the same time point, thymocytes from PD rats presented lower IkB levels (42%) in the cytoplasm when compared to controls. The leptin incubation (24 h) had no effect upon either control or PD groups.

### Maternal Protein Deprivation Increases Leptin Gene Expression in the Thymic Microenvironment of Pups

**Figure 7:**
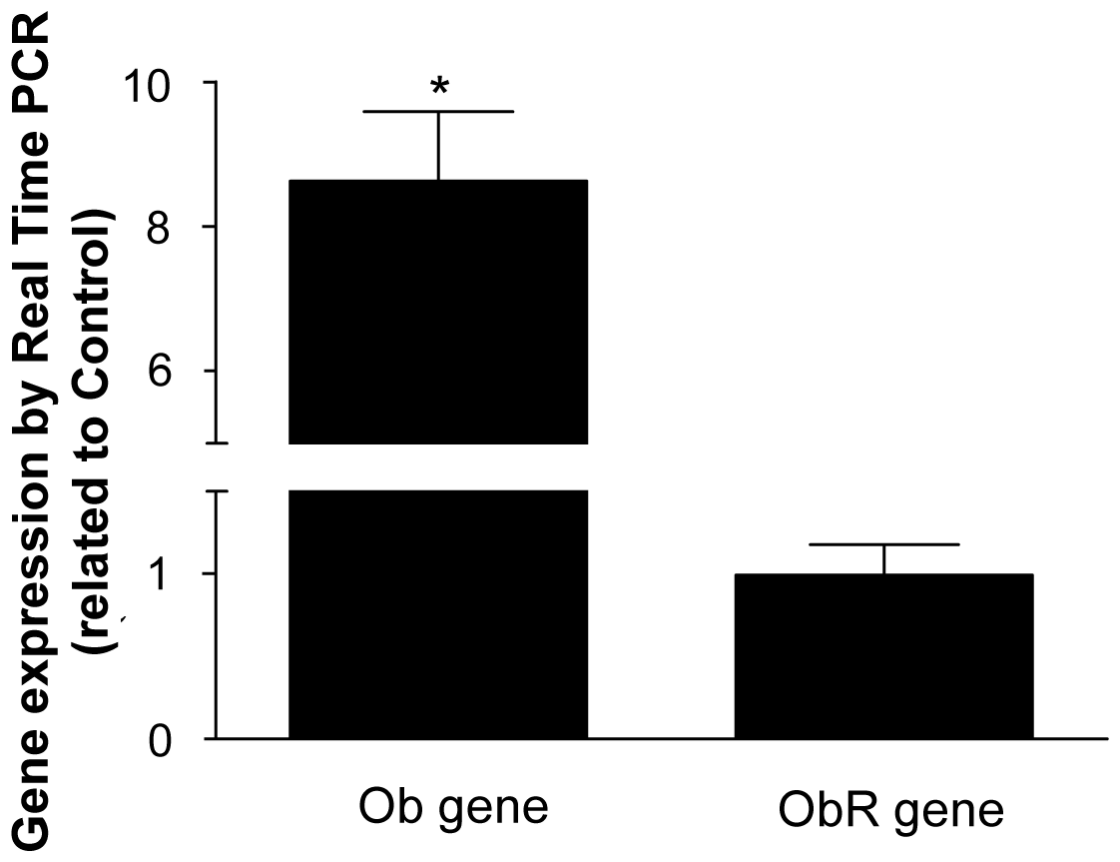
Thymic microenvironment isolated from PD rats presents higher Ob gene expression levels. Total RNA was harvested and analyzed by qRT-PCR for Ob and ObRb genes mRNA. The results were normalized by GAPDH. The results were expressed as expression related to control. Values are means ± SE of 6 animals/group. *P<0.05 compared to control group.

Given that circulating leptin levels at 25^th^ and 30^th^ day are similar in PD and control groups, we next asked whether the intrathymic production of the hormone by thymocytes and/or thymic microenvironmental cells could be modulated secondary to maternal protein deprivation. For that we evaluated leptin gene expression in thymocyte and microenvironmental cell preparation from control and PD rats. We showed that the thymic microenvironment isolated from PD group presented a higher mRNA level of *Ob* gene when compared to control group (Fig. 7). We also evaluated the *Obrb* gene expression in the thymic microenvironment of these animals and we observed similar mRNA levels of the leptin receptor in both groups (Fig. 7).

## Discussion

Nutritional stress during critical developmental periods is accompanied by significant metabolic and endocrine changes [6], [8], [10] that could potentially contribute to alterations on immune response [10], [11]. We are now providing evidence that maternal protein deprivation during early lactation in rats induces permanent alterations in leptin signaling in thymus of the young progeny, which seems to affect the apoptotic program in thymocytes.

The mild maternal protein restriction during lactation was already shown to induce a significant reduction in body weight of young offspring (12), and we have demonstrated that occur a proportional reduction in the thymus weight. However, this effect was not accompanied by severe alterations in offspring’s thymus cellularity. We also performed histological analysis of thymus from control and PD animals by haematoxylin-eosin staining. We observed that histological structure of PD thymus was clear-cut identical from control thymus (data not shown). These results suggest that other factors would be involved in protection the thymus from massive apoptosis.

The interference of malnutrition on the survival of thymic cells was early reported. Increased spontaneous apoptotic activity in thymocyte associated to severe malnutrition was observed in rats imposed to malnutrition by food competition during lactation [37], and also in malnourished children [38], [39]. Conversely, our results indicated a protection of thymocyte from apoptosis in the progeny of dams submitted to a mild protein restriction during lactation.

The tight connection between thymic function in malnutrition and leptin activity in this tissue has been highly demonstrated, reinforcing the role of this hormone in the connection between metabolic and immune function [29], [40], [41]. Indeed, both, animals and humans, with defect in the leptin functions, present features of immunodeficiency, including thymic atrophy [29]. Maternal dietary alterations during perinatal periods were shown to affect leptin sensitivity in offspring [42]–[44]. Furthermore, Lisboa and co-workers, using the same animal model that we have used, demonstrated an over-expression of leptin receptor in the pituitary gland in adult rats [45].

Our results show that PD rats present at the last day of lactation (21 days of age) increased serum leptin that rapidly decreased to control levels after weaning, at 25 and 30 days. As leptin is present in the milk, its higher levels at the 21^st^ day may be due to its transference to the pups through the milk of malnourished mothers [12]. On the other hand, despite the normal leptin secretion, thymocytes from young PD rats presented a higher ObR protein expression, but no significant variation in the mRNA levels for *Obrb*. The data suggest that ObR protein expression in thymocytes of PD animals may be regulated by a post-transcriptional mechanism. Considering that we have also demonstrated that thymic cells from PD rats presented an increased signaling response to leptin, we suggest that the maternal protein deprivation during lactation can lead to an increase in the sensitivity of thymocytes to leptin-induced signaling in offspring.

Recent studies already demonstrated that leptin promotes hematopoietic processes to include lymphopoiesis and myelopoiesis. Ob/ob mice produce a nonfunctioning leptin, and despite their large size, they present thymic atrophy and depleted hematopoietic compartments. In these mice, the treatment with recombinant exogenous leptin rejuvenates the depleted primary tissues [46]. In our experimental model, the results suggest that serum leptin at 30 days of age does not modulate hematopoietic processes as lymphopoiesis, because we did not observed alterations in the thymus weight or in the total number of thymocytes. Others studies also demonstrated that relation between leptin and thymopoiesis is positively correlated only in ob/ob mice (deficient state of leptin). In wild type mice, leptin did not stimulate thymopoiesis but significantly lowered thymus weight [47], [48].

We also found a significant increase in the relative numbers of CD4+ and CD8+ mature thymocytes in the young PD offsprings. It has been postulated that leptin can significantly affect the differentiation of immature T cells inducing a more mature phenotype [47], [49], [50]. The profile seen in our model may be related to higher leptin sensitivity observed in the thymocytes from PD rats. In fact, it has been showed that incubation of thymocytes with leptin induce CD4+CD8+ cell differentiation mainly to CD4+ mature thymocytes [51].Within the spleen, the expression of these membrane surface membranes were unchanged. It suggests that maternal protein restriction during lactation does not affect this cell population.

It has been demonstrated that intrauterine growth restriction can induces apoptosis in fetal kidney and in beta cells of pups compromising the adult renal function and insulin secretion, respectively [52], [53]. Nevertheless, the maternal protein restriction during lactation had a protective effect on the thymocytes with lower spontaneously apoptosis rate, without altering the proliferative response. Once again, these results could be explained by an increase in leptin activity. In particular, leptin acts directly to inhibit the thymocytes apoptosis [28], [30], [54], which is in accordance with our data showing that, after leptin incubation, thymocytes were clearly protected from apoptosis.

It has been demonstrated that the inhibitory effect of leptin on the thymocytes apoptosis does not involves the classical pathway activation [30]. Our findings are consistent with this report by showing that, in PD offspring, the capacity of leptin in inhibiting thymocytes apoptosis was dependent of ObRb, though independent of JAK2 actvation. Therefore, we hypothesized the possibility that PI3K, IRS1 or ERK2 could be mediate the leptin protective effect in thymocytes from PD rats however, this possibility was excluded by detecting no difference in neither AKT, IRS1 or ERK-2 activation in basal or leptin stimulated conditions (data not shown). Further investigations will be helpful to understand the mechanism involved in this effect.

Leptin-related apoptosis inhibition might then be associated to changes in the expression balance of pro-and anti-apoptotic proteins. It has been addressed the role of BCL2 family members in the T-cell development and different types of thymocyte programmed cell death [55]–[58]. BCL2 overexpression protects thymocytes from multiple apoptotic stimuli [59] and it is expressed at high levels in mature thymocytes and peripheral T cells [60], being also an inhibitor of pro-apoptotic BCL2 family members, such as BAX. Moreover, BAX transgenic thymocytes exhibited an accelerated apoptosis in response to dexamethasone [61] and an *in vitro* study has showed the involvement of BAX and CASPASE3 in the 5-fluoracil-induced apoptosis on murine thymocytes [62]. Our results confirmed these hypotheses, demonstrating that maternal deprivation during lactation induces BCL2 and reduces BAX expression, in addition to inhibiting CASPASE3 activation in thymocytes, ultimately resulting in an anti-apoptotic phenotype.

Transcription of anti-apoptotic molecules, such as BCL2 is regulated by NF-kB transcriptional activation. All thymocytes subsets present some constitutive NF-kB expression and, notably, *ex vivo* NFkB inhibition in isolated DN thymocytes triggers apoptosis [63] suggesting that NF-kB provides a survival signal. Nevertheless, few studies describe the effects of leptin on NF-kB activation. Bouloumié and col (1999) demonstrated that leptin induces activation of both AP-1 and NF-kB in HUVEC [64]. Herein, our results showed a link between the decreased spontaneous apoptosis rate observed in PD thymocytes and the increased NF-kB activation, associated to lower IkB contents in the cytoplasm. Interestingly, an up-regulation of the NF-kB system has been revealed in neutrophils isolated from adult offspring from dams submitted to severe (0% protein) protein deprivation during early lactation [11]. However, in our experiments leptin seems unable to significantly induce nuclear translocation of NFkB in thymocytes from control group. This discrepancy may be related to time course (24 hours) of our experiment. It has already been demonstrated that NFkB -binding activity presents a biphasic profile in caput epididymis from rats after LPS treatment, with a first peak at 2 hours and a second one at 15 hours, returning to baseline levels in 24 hours [65].

The effects on thymocyte survival might also be related to alterations on the glucocorticoid secretion and activity. We have previously showed that adult rats submitted to severe maternal protein deprivation (0% protein) during the first ten days of lactation have high levels of circulating glucocorticoids [10]. It has been known that glucocorticoids are very potent inducers of thymocytes apoptosis [66]–[69] acting through interaction with the corresponding GR receptor [70]. However, in the present study, the serum corticosterone levels remained unaltered in young rats from PD group, when compared to controls. Additionally, analysis from nuclear contents suggested that there are no alterations in the GC activity in the thymocytes from PD group (data not shown). Thus, it does not seem likely that a glucocorticoid-mediated circuit is involved in the effects we have described.

Interestingly, at 25 days of age, we observed no difference in the mRNA expression for ob gene between the control and PD groups (data not shown). However, at 30 days of age, PD rats presented a higher expression of mRNA for *Ob* gene in the thymic microenvironment. These findings, together with the bulk of the data on thymocytes, indicate that an intrathymic paracrine leptin-mediated circuit may be altered in young adult offspring due to the maternal protein deprivation during lactation, protecting thymocytes from apoptosis, by regulating key apoptosis-related proteins, without altering proliferation.
